# Predictive Modeling of Physician-Patient Dynamics That Influence Sleep Medication Prescriptions and Clinical Decision-Making

**DOI:** 10.1038/srep42282

**Published:** 2017-02-09

**Authors:** Andrew L. Beam, Uri Kartoun, Jennifer K. Pai, Arnaub K. Chatterjee, Timothy P. Fitzgerald, Stanley Y. Shaw, Isaac S. Kohane

**Affiliations:** 1 Department of Biomedical Informatics, Harvard Medical School, Boston MA, USA; 2 Center for Systems Biology; Center for Assessment Technology & Continuous Health (CATCH), Massachusetts General Hospital, Boston MA, USA; 3 Harvard Medical School, Boston MA, USA; 4 IBM Research, Cambridge MA, USA; 5 Merck & Co., Inc., Boston, MA, USA.; 6 Merck & Co., Inc West Point, PA, USA.

## Abstract

Insomnia remains under-diagnosed and poorly treated despite its high economic and social costs. Though previous work has examined how patient characteristics affect sleep medication prescriptions, the role of physician characteristics that influence this clinical decision remains unclear. We sought to understand patient and physician factors that influence sleep medication prescribing patterns by analyzing Electronic Medical Records (EMRs) including the narrative clinical notes as well as codified data. Zolpidem and trazodone were the most widely prescribed initial sleep medication in a cohort of 1,105 patients. Some providers showed a historical preference for one medication, which was highly predictive of their future prescribing behavior. Using a predictive model (AUC = 0.77), physician preference largely determined which medication a patient received (OR = 3.13; p = 3 × 10^−37^). In addition to the dominant effect of empirically determined physician preference, discussion of depression in a patient’s note was found to have a statistically significant association with receiving a prescription for trazodone (OR = 1.38, p = 0.04). EMR data can yield insights into physician prescribing behavior based on real-world physician-patient interactions.

Insomnia remains under-diagnosed and poorly treated despite its high economic and social costs^1^. Prevalence estimates for insomnia are reported to be as high as 40% depending on the specific study definition^2^. Studying insomnia is complicated by an ambiguous clinical definition, as it is often considered both symptom and disease. This nosological fluidity may result in incomplete or inconsistent documentation in traditional research resources, such as the billing codes found in insurance claims databases, and further challenges efforts to understand the epidemiology and treatment of insomnia.

Here we focus on pharmacologic treatment decision making using a fully data-driven probabilistic approach to characterize insomnia and its comorbidities. The treatment of insomnia includes behavioral/psychological interventions (stimulus control therapy, relaxation therapy, paradoxical intention, sleep restriction, cognitive behavioral therapy) and/or pharmacotherapy (benzodiazepines, non-benzodiazepines, melatonin, antidepressants, anti-epileptics, and antipsychotics); however, a large number of these agents are used off-label due to their sedative effects^3^,^4^. Current pharmacotherapy is associated with next-day sedation/somnolence, psychomotor impairment, and cognitive impairment and common adverse effects associated with pharmacotherapy include headache, drowsiness, fatigue, nausea, vomiting, paresthesia, incoordination, dizziness, hallucinations, and ataxia^3^,^4^. As a result of these adverse effects, although treatment can improve mental quality of life, it may actually decrease physical quality of life in patients with insomnia^3^,^5^.

Many sleep-medications, such as zolpidem (Ambien) and eszopiclone (Lunesta), have been shown to be effective for both transient and chronic insomnia management, but come with a long list of potential side effects and warnings^6^,^7^. A popular alternative is off-label usage of the anti-depressant trazodone, which is perceived to have fewer side effects but has less evidence of its effectiveness as a sleep aid^8^. How physicians decide which of these medications to prescribe in response to insomnia remains poorly understood. Previous studies^9^,^10^ have sought to understand the patient characteristics that underlie the prescription patterns for patients with insomnia. For example, one study^11^ found that patients experiencing depression were more likely to receive an off-label antidepressant to treat their insomnia. The rationale is that the physician is responding to the patient’s dual-state of depression and insomnia and that the anti-depressant could treat both. However, it is unclear to what extent “physician-patient dynamics” (i.e. the characteristics of both physicians and patients) influence clinical decision-making.

In this study, we seek to understand the physician-patient dynamics that influence how sleep medications are prescribed. Traditionally, physician prescribing behavior is studied using market surveys of physicians^12^ but these are expensive and labor-intensive to undertake. Similarly, databases of prescriptions, insurance claims or drug advertising^12^,^13^ are commonly analyzed but these provide a severely restricted, incomplete view into the patient-physician encounter. Unique to our study is the perspective that both physician and patient factors are important contributors to clinical decision-making. Electronic medical records (EMRs) present a valuable resource to interrogate prescription patterns because both codified data (prescriptions, billing codes for insomnia and comorbid diseases) and narrative data (physician office notes) may be analyzed. Furthermore, detailed data about both the physician and patient can be linked in our analysis: the EMR used in this study provides identifiers for each patient and physician during a clinical encounter. Using this linkage, we can decompose a clinical decision to better understand patient-specific (e.g. conditions that are comorbid with insomnia) and physician-specific (e.g. a historical preference for a given medication) attributes that drive clinical decision-making. Understanding the duality of this physician-patient interaction is novel and unique to our study, and may be more broadly useful for studies of medication prescribing patterns in conditions other than insomnia.

We study the time period surrounding when a patient is diagnosed with insomnia and is given their first prescription for a sleep medication in the outpatient setting. Our central hypothesis is that the patient and provider both bring elements to this encounter that may influence the final decision. Through a novel combination of machine learning and natural language processing (NLP), we extracted information on the comorbid conditions of the patient and the providers’ historical preference for a particular sleep medication, and weigh these two elements against one another to gain a deeper understanding of how the decision was made.

## Results

### Distribution of Initial Medication Prescriptions

We identified 1,105 patients (the START cohort) that were receiving their first documented prescription for a sleep medication. Demographic and comorbidity characteristics for this cohort are presented in Supplementary Table 1. Diagnosis and procedure codes used to define the comorbidities are presented in Supplementary Table 2.

**Figure 1:**
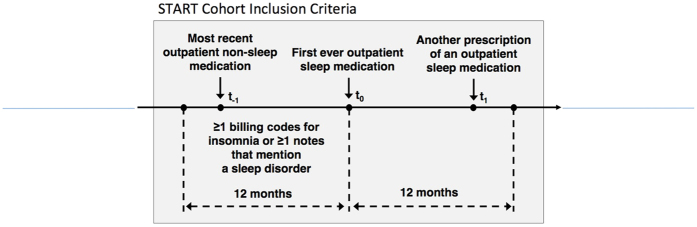
START cohort inclusion criteria: Patients receiving their first prescription for sleep medication in response to a documented case of insomnia or a sleep disorder. For inclusion, a patient must have an indication of insomnia (e.g., billing code or mention in a note) and have no previous record of a sleep medication before *t*_*0*_ and receive another sleep medication in the subsequent 12-month period indicating continued use.

**Figure 2:**
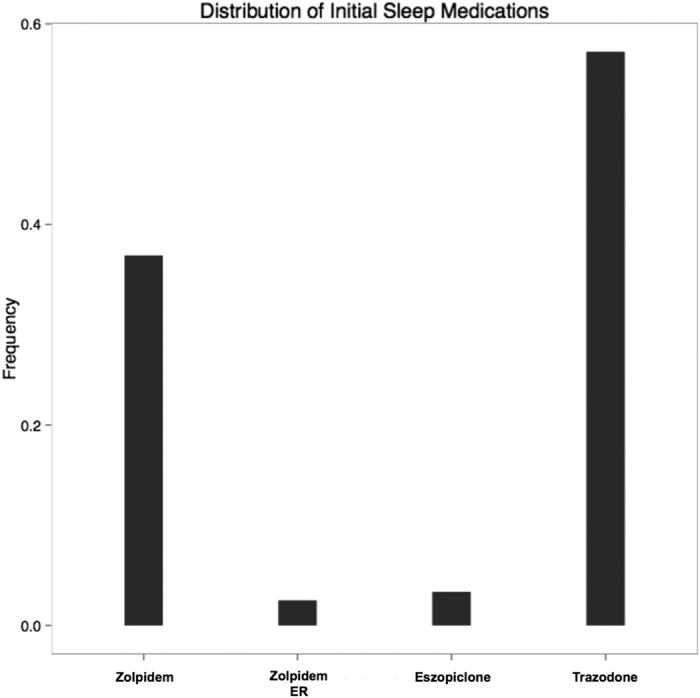
Distribution of the initial sleep medication prescriptions for the START cohort. Each bar represents the fraction of patients that were prescribed a given medication. Trazodone was the mostly widely prescribed with zolpidem being a popular second choice. Eszopiclone and zolpidem extended release (ER) were distant third and fourth choices, respectively.

We extracted clinical narratives from the *t*_*0*_ time period shown in Fig. 1. These notes are from the 14-day window (+/−7 days) surrounding the encounter where the sleep medication was prescribed. The distribution of the medication patients received as their initial sleep medication is summarized in Fig. 2.

Trazodone was prescribed to roughly 57% of the patients in the START cohort, while zolpidem was prescribed to nearly 38%. Zolpidem extended release and eszopiclone were both prescribed to fewer than 5% of patients in this cohort. Since trazodone and zolpidem made up 90% of the prescriptions, we focused exclusively on these two medications for the remainder of this investigation. Note: that these are mutually exclusive categories; each patient in the START cohort received one and only one medication. Since age and gender could potentially influence which medication a patient may receive, we first investigated if there were any differences we between these two demographic variables. We found there were no differences in age (p = 0.74, t-test) or gender (p = 0.64, Fisher’s exact test) between the trazodone and zolpidem populations.

### Historical Physician Preference for Trazodone vs. Zolpidem

**Figure 3:**
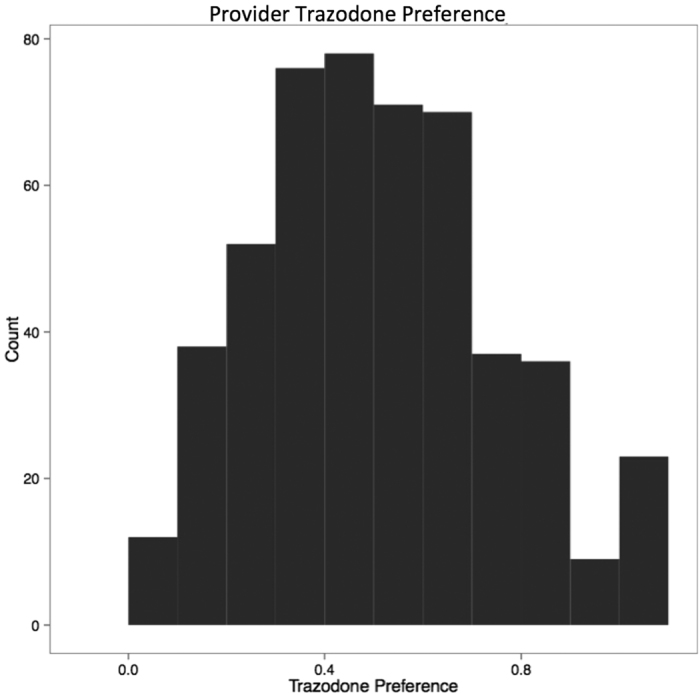
Distribution of the provider preference, *p*_*i*_, for trazodone over zolpidem. Note the small spike at *p*_*i*_ = 1, indicating a small fraction of providers prefer trazodone exclusively.

Next, using the historical prescribing behaviors for the physicians in this cohort, we derived the trazodone preference (see Methods) for each provider in the START cohort. Figure 3 displays the distribution of trazodone preference in our physician cohort. Note that there is a small spike at 1, indicating that some physicians in this cohort exclusively prescribe trazodone.

### Prediction of Initial Sleep Medication

Next, we combined the provider preference variable with the topic variables extracted from the notes using Latent Dirichlet Allocation (LDA)^14^ and fit a logistic regression model for a varying number of topics (K) as the number of topics is a tuning parameter in our model. We employed the topic modeling approach to explicitly capture the distinct groups of patients as defined by the clinical narratives of their doctors rather than an arbitrary categorization of patients by external experts. Each prescription has an associated timestamp in the EMR. To avoid a model that over-fits, we computed a time-specific provider preference for each encounter. For instance, if a patient received a prescription on January 1^*st*^, 2010 the provider preference was computed using only prescriptions that occurred before this date. For the values of K = {0, 5, 10, 15, 20}, we estimated the area under the receiver-operator curve (AUC) using 10 fold cross-validation. The estimates from this process are shown in Table 1. We fit a model with the number of topics equal to 0 to estimate the value added by incorporating the information from the notes relative to the provider preference. The top-performing model was for K = 5, so we continued with this model for further analysis. Next, we examined the odds ratios and p-values from the best model to see which variables were the most informative. This information is displayed in Table 2.

**Figure 4:**
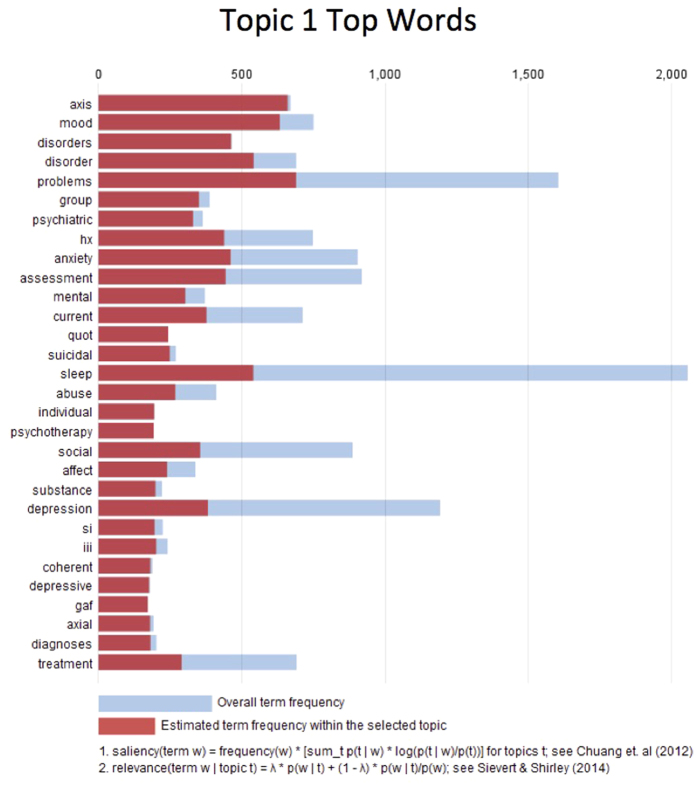
Visualization of top words from Topic 1. These are the top 20 words that are most associated with Topic 1. Inspection reveals that this topic most likely describes depression and psychiatric disorders. The odds-ratio (OR = 1.38, p = 0.04) indicates that patients that have a note with this topic are more likely to be prescribed trazodone, as the physician may be responding to both the insomnia and depression.

The variable for provider preference was the most significant variable in the model, measured both by the effect size and p-value (OR = 3.13; p = 3 × 10^−37^). An OR > 1 for provider preference indicates that if a physician has a higher historical preference for trazodone, there is a higher probability that he or she will prescribe a new patient trazodone as their first sleep medication. Additionally, Topic 1 produced by LDA was found to have a statistically significant odds-ratio (OR = 1.38, p = 0.04) for receiving trazodone. In Fig. 4, we have visualized the top words for Topic 1, to better understand what this topic might be describing. The figure shows the top 30 words that are specific to Topic 1. Several informative words appear including “depression”, “mood”, “psychiatric”, “mental”, “suicidal”, and “substance” indicating this topic most likely refers to depression and anxiety. Since this variable was found to be important, it could indicate that providers are responding partially to a depressed patient’s clinical state and are prescribing them trazodone to treat both insomnia and depression. The AUC for this model estimated using 10-fold cross-validation was 0.77, indicating the model contains significant explanatory power for this clinical decision.

Additionally, we fit a logistic regression model without the trazodone preference variable with K = 5 LDA topics and observed an AUC = 0.54 in 10-fold cross-validation. The topic indicating depression (Topic 1) was again significant (OR = 1.4, p = 0.015) in this scenario when trazodone preference was omitted, though all other topics again failed to achieve statistical significance. Taken together, the variable odds-ratios and model AUC indicate that provider preference, relative to patient specific factors, is likely the major determining factor driving which sleep medication a patient receives, though in some instances the provider may be responding to certain characteristics of the patient such as depression.

## Discussion

There is a growing awareness that clinical decisions do not occur in a vacuum and are subject to numerous social and psychological forces. For instance, a previous study found that provider prescribing habits can be influenced by other physicians in their social network^12^. Another study found that some providers routinely prescribe a reduced subset of available drugs relative to their peers, and that this reduced list most often contained drugs that were heavily advertised^15^. These are representative studies of prescribing behavior in that they utilized marketing surveys^12^ or structured databases^12^,^15^. Commonly interrogated databases (e.g., prescriptions, claims, and advertising) can indirectly reflect prescribing behavior, but lack the information needed to understand the clinical decision-making process or patient opinions.

In this study we a present new methodology for understanding the physician-patient dynamics that drive clinical decisions. Using this perspective, we developed a machine-learning and NLP-based predictive model to dissect the various physician-patient factors involved in sleep medication prescriptions. Further development of this framework may be applied more broadly to medication prescribing patterns for other challenging conditions. Using this approach, we found that a provider’s historical preference of medication was the dominating factor for determining which sleep medication a patient received. Nonetheless, there was a statistically significant association with having a note discussing depression and the likelihood that a patient would receive trazodone. There likely are clinical domains where either the differential efficacy and/or the education about the effectiveness of alternative drugs, or patient-specific factors, leads to greater variation in drug prescription patterns for individual physicians. We believe this study could be of clinical significance as it sheds light on an area that is often understudied (insomnia) and highlights an opportunity for further physician education on medication prescriptions for this patient population.

Additionally, this study reinforces the importance of using both billing codes and unstructured notes for the study of insomnia. Because insomnia is often incompletely documented, the billing code record may only partially identify patients experiencing insomnia. In our study, only 476 of the 1,105 patients in the START cohort had at least one billing code for insomnia, thus we were able to identify additional 629 patients through the use of the clinical notes. Taken together, these results suggest that analysis of structured and narrative EMR data can illuminate specific aspects of physician and patient decision-making in a manner inaccessible to traditional study methods, and enhance our understanding of physician-patient dynamics in healthcare.

## Methods

### Cohort Selection

We analyzed a previously defined cohort of 314,292 patients at increased risk for metabolic syndrome who attended Massachusetts General Hospital (MGH) or Brigham and Women’s Hospital (BWH) between 1992 and 2010^16^. Patients in this cohort had at least one type 2 diabetes mellitus (T2DM) diagnosis code, or a T2DM medication, or HGB A1C ≥ 6.5%, or plasma glucose ≥ 200 mg/dl.

We selected for our study cohort a set of patients seen at Partners HealthCare system with either confirmed diabetes or a suspicion of diabetes. We selected this group out of several other cohorts because A) the prevalence of diabetes is so high in Boston that the numbers of such patients that we could obtain was high and B) patients with diabetes manifest a range of co-morbidities related to both cardiovascular disease and cancer, such that we would have a very rich set of medical conditions under which we could examine physician prescribing behavior. Also because the treatment of diabetes and its complications involve a large number of sub-specialties as well as primary doctors, we would have the ability to study the range of behaviors across a heterogeneous mix of physicians.

From this population, we identified a cohort of patients that correspond to a clinical decision point involving a prescription for a sleep medication that occurred in an outpatient setting. The cohort (Fig. 1), hereafter referred to as the START cohort, consisted of patients receiving a medication prescription in response to a documented diagnosis of insomnia. Patients in this cohort have at least one indication for insomnia in the form of an International Classification of Diseases (9^*th*^ Revision) (ICD-9) code (307.41, 307.42, 327.00, 327.01, 327.02, 327.09, 780.52) or a mention of a sleep disorder in a note in the 12-month period before receiving their prescription for a sleep medication. Expressions representing a sleep disorder include: “poor sleep,” “has trouble sleep,” “reduced sleep,” “increased sleep,” “decreased sleep,” “excessive sleep,” “fragmented sleep,” “sleeplessness,” “sleep disruption,” or “sleeps poorly.” Additionally, these patients must have had no prior entry for a sleep medication in their EMR, which implied that these patients were receiving the first such prescription for a sleep medication. The START cohort inclusion criteria are summarized in Fig. 1.

Additionally, we developed a physician cohort composed of every physician that saw a patient in the START cohort. In order to learn about the historical medication preferences of these physicians, we retrieved every previous prescription written by the physician that was available in the EMR. In total there were 504 physicians included in this group.

### Statistical Methodology

We pulled the entire prescription history for every provider that saw a patient in the START cohort. Next, we defined a provider specific preference for trazodone vs. zolpidem, *p*_*i*_, as:


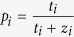


where *t*_*i*_ is the total number of prescriptions written by the provider for trazodone and *z*_*i*_ is the total number of prescriptions written by the provider for zolpidem. This quantity captured a provider’s relative preference for prescribing trazodone or zolpidem as a sleep medication; the minimum value of 0 indicated an exclusive preference for zolpidem while the maximum value of 1 indicated an exclusive preference for trazodone.

To summarize the information available in each note, we used a machine learning technique known as Latent Dirichlet Allocation (LDA)^17^. LDA is a hierarchical Bayesian model that discovers topics in text documents by clustering words that co-occur frequently together and then models each document as a distribution over these discovered topics. LDA provides high-level and coherent summaries of the information and has been shown to be an accurate representation of the content of clinical notes^18^. The topics discovered by LDA can have meanings that are interpretable by humans^19^ (though is not always the case), making it an useful tool for modeling clinical narratives. The number of topics to include in a model is a free parameter that must be chosen, similar to the number of clusters in k-means clustering. We fit LDA models with K = {5, 10, 15, 20} topics for use in the next modeling stage.

Next, we used topic probabilities from LDA and provider preference as independent variables in a logistic regression model to predict which medication (trazodone or zolpidem) would be given as the initial sleep medication. Specifically, for each patient note we have K probabilities representing the degree to which each topic is present in the note and the historical provider preference up to the date of the encounter. Note that the probabilities from LDA form a multinomial distribution over topics and thus sum to 1, so any one probability is perfectly determined from the other K-1 probabilities (i.e. probability K is equal to 1 minus the sum of the other (K-1 probabilities). This colinearity will produce an error when fitting a logistic regression model (due to a matrix singularity) so without loss of generality, we drop the last topic K (i.e. set the regression coefficient for that topic to 0) from the design matrix when fitting the logistic regression model. Under this scheme, coefficient for topic K is absorbed into the intercept and will serve as the reference level for the remaining topics. This is similar to how categorical variables are often represented where one level of the variable is set as the reference group. For each value of K = {0,5, 10, 15, 20}, we estimated the area under the receiver-operator curve (AUC) using 10-fold cross-validation. The topics were visualized with LDAvis^20^ and words were ranked according to their *relevance* as defined in the LDAvis manuscript with lambda = 0.6.

We defined *p* = 0.05 as the significance threshold. All programming was performed using the R statistical language^21^.

This study and all study methods including EHR cohort assembly, data extraction and analysis were approved by the institutional review board (IRB) of Partners Healthcare.

## Additional Information

**How to cite this article**: Beam, A. L. *et al*. Predictive Modeling of Physician-Patient Dynamics That Influence Sleep Medication Prescriptions and Clinical Decision-Making. *Sci. Rep.* **7**, 42282; doi: 10.1038/srep42282 (2017).

**Publisher's note:** Springer Nature remains neutral with regard to jurisdictional claims in published maps and institutional affiliations.

## Supplementary Material

Supplementary Information

## Figures and Tables

**Table 1:** Performance metrics for the logistic regression model for various values for the number of topics extracted using LDA.

Number of Topics	AUC(SD)	Sensitivity (SD)	Specificity (SD)
0	0.764 (0.055)	0.769 (0.045)	0.656 (0.570)
5	0.769 (0.057)	0.759 (0.050)	0.658 (0.090)
10	0.762 (0.040)	0.755 (0.036)	0.652 (0.069)
15	0.757 (0.035)	0.750 (0.085)	0.650 (0.062)
20	0.747 (0.063)	0.751 (0.057)	0.656 (0.064)

AUC, Sensitivity, and Specificity are given with standard deviations (SD). The first row (number of topics = 0) displays results from a logistic regression model fit using only the variable for provider preference.

**Table 2:** Odds ratios of each variable for the best cross-validated model containing provider preference and the LDA topics.

Variable	Odds Ratio	p-value
Intercept	0.95	0.46
Provider Preference	3.13	3 × 10^−37^
Topic 1	1.38	0.04
Topic 2	1.00	0.97
Topic 3	1.22	0.14
Topic 4	1.23	0.24
