# Supplementary material for: Predictive Modeling of Physician-Patient Dynamics That Influence Sleep Medication Prescriptions and Clinical Decision-Making
Source: Sci Rep. 2017 Feb 9;7:42282. doi: 10.1038/srep42282 (PMC5299453; doi:10.1038/srep42282)
Supplement: Supplementary Information [file srep42282-s1.doc]

**Supplementary Information accompanying the manuscript:**

**Predictive Modeling of Physician-Patient Dynamics That Influence Sleep**

**Medication Prescriptions and Clinical Decision-Making**

**Andrew L Beam PhD^1,2^*, Uri Kartoun PhD^2,3^*, Jennifer K Pai ScD MHS^4^, Arnaub K Chatterjee MHA MPA^4^, Timothy P Fitzgerald PhD^4^,** **Stanley Y Shaw MD PhD^2,3^*, Isaac S Kohane MD PhD^1,2^***

1. Department of Biomedical Informatics, Harvard Medical School, Boston MA.

2. Center for Systems Biology; Center for Assessment Technology & Continuous Health (CATCH),

Massachusetts General Hospital, Boston, MA.

3. Harvard Medical School, Boston, MA.

4. Merck & Co., Inc.

**Supplementary Table 1 - Characteristics of the START cohort.** Patients were identified by having either ≥1 instances of the ICD-9 code for insomnia or having ≥1 notes that mention a sleep disorder within the preceding 12 months to the first sleep medication. A patient is counted as having the comorbidity if he or she is associated with ≥1 procedure codes or ≥2 ICD-9 codes from date of birth to the first prescription of a sleep medication. Only comorbidities with prevalence of ≥10% are presented.

| **Variable and category** | **Insomnia patients**  **(n = 1,105)** |
| --- | --- |
| **Mean (SD) age (years)** | 54.7 (15.8) |
| **Gender (%):** | |
| Male | 40.2 |
| Female | 59.8 |
| **Ethnicity (%):** | |
| White | 66.1 |
| African American | 13.2 |
| Other | 0.5 |
| Unknown | 20.3 |
| **Marital Status (%):** | |
| Married or partner | 42.7 |
| Other | 55.7 |
| Unknown | 1.6 |
| **Comorbidities (%):** | |
| Joint disorder | 62.9 |
| Hypertension | 58.0 |
| Disorders of lipid metabolism | 50.5 |
| Diabetes | 39.3 |
| Gastronomical disorder | 32.4 |
| Psychiatric disorder | 30.7 |
| Anxiety or depression | 30.6 |
| Obesity | 23.7 |
| Asthma | 18.5 |
| Pneumonia | 14.8 |
| Coronary artery disease | 13.8 |
| Cancer | 12.4 |
| Chronic obstructive pulm. disease | 12.3 |
| Congestive heart failure | 11.9 |
| Cerebrovascular disease | 11.0 |
| Sleep apnea | 10.1 |

**Supplementary Table 2. Diagnosis, procedure, and CPT codes used to define comorbidities**

| **Comorbidity** | **ICD9 Diagnosis Codes** | **ICD9 Procedure Codes** | **CPT Codes** |
| --- | --- | --- | --- |
| **Alzheimer’s disease / dementia** | 331.0, 294.1  Dementia: 290, 294.1 | - | - |
| **Anxiety or depression** | 296.2, 296.3, 300.xx | - | - |
| **Asthma** | 493.xx | - | - |
| **Atrial fibrillation /**  **Atrial flutter** | 427.31, 427.3, 427.32 | 37.33, 37.34 | 93653 to 93657, 33254 to 33259, 33265 to 33266 |
| **Cancer** | Breast cancer: 174.xx  Colon cancer: 153.xx  Esophageal cancer: 150.xx  Pancreatic cancer: 157.xx  Prostate cancer: 185.xx  Renal cancer: 189.xx  Thyroid cancer: 193.xx  Gall bladder cancer: 156.xx  Uterine cancer: 179.xx  Cervical cancer: 180.xx  Lung cancer: 162.xx | - | - |
| **Cerebrovascular disease** | 430.xx, 431.xx, 432.xx, 433.xx, 436 | - | - |
| **Chronic kidney disease / end stage renal disease** | 585.1, 585.2, 585.3, 585.4, 585.5, 585.6, 585.9 | - | - |
| **Cirrhosis** | 571.2, 571.5, 571.6 | - | - |
| **Congestive heart failure** | 428.xx |  |  |
| **COPD** | 491.xx, 492.xx, 496 | - | - |
| **Coronary artery disease (ischemic heart disease / myocardial infarction)** | 410, 411, 412, 413, 414, 410.xx | 36, 00.66 | 92995, 92996, 92982, 92984, 92980, 92981, 33510 to 33545 |
| **Diabetes** | 250.xx | - | - |
| **Disorders of lipid metabolism (dyslipidemia, hyperlipidemia, and related others)** | 272, 272.0, 272.1, 272.2, 272.3, 272.4, 272.5, 272.6, 272.7, 272.8, 272.9 | - | - |
| **Gastrointestinal disorder** | Duodenal ulcer: 532.xx  Esophageal reflux: 53081  Esophagitis: 5301  Gastric ulcer: 531.xx  Gastritis and Duodenitis: 535.xx  Gastrojejunal ulcer: 534.xx  Peptic ulcer: 533.xx  Ulcer of esophagus: 5302 | - | - |
| **Hypertension** | 401.xx, 997.91 | - | - |
| **Joint disorder** | Crystal arthropathies: 712.xx  Rheumatoid arthritis and other inflammatory Polyarthropathies: 714.xx  Dorsopathies: 720.xx, 721.xx , 722.xx , 723.xx , 724.xx  Internal derangement of knee: 717.xx  Osteoarthrosis and allied disorder: 715.xx  Psoriatic arthropathy: 696.0 | - | - |
| **NAFLD** | 571.7, 571.8 | - | - |
| **Non-viral hepatitis** | 571.4, 571.6, 571.9, 571.0, 571.2, 571.3, 571.1, 571.42, 573.8, 965.4 | - | - |
| **Obesity** | 278, 278.0, 278.00, 278.01, 278.02 | - | - |
| **Osteoporosis** | 733.0x | - | - |
| **Peripheral vascular disease** | 443.9, 785.4, 250.7 443.81, 440.21, 440.22, 440.23 | 84.1x, 00.55, 39.90, 00.60, 39.22, 39.24, 39.25, 39.26, 39.50, 38.13, 38.18, 00.40 to 00.43, 00.46 to 00.48 | 37220, 37222, 37224, 37228, 37232, 37205, 37221, 37223, 37226, 37230, 37234, 27590 to 27598, 28800, 28805, 28810, 28820, 28825, 27880, 27881, 27882, 27884, 27886, 28888, 27889, 27290, 27295 |
| **Pneumonia** | 480.xx, 481.xx, 482.xx, 483.xx, 484.xx, 485.xx, 486.xx, 487.0, 488.01, 488.11 | - | - |
| **Psychiatric disorder** | 294.0, 294.8, 294.9, 295.xx, 296.xx, 297.xx 298.xx (excluding 296.2, 296.3) | - | - |
| **Renal failure** | 250.4, 585, 583.81, 581.81 | 00.91, 00.92, 00.93, 39.95, 54.98 | 90935, 90937, 90945, 90947, 90999, 50360, 50365 |
| **Sleep apnea** | 327.23, 327.2, 327.29, 780.57, 327.20, 327.21, 780.51, 780.53 | - | - |
| **Stroke** | 434.91 | - | - |
| **Viral hepatitis** | 070.xx | - | - |
